# From “Preposition”

**Published:** 1886-01

**Authors:** 


### Communications to the Editor.

BUFFALO, Dec. 27, 1885.

*Editors of the Journal:*

One of the most agreeable to meet, and certainly the most contented-looking man seen at the late meeting of the New York State Medical Association, was the President, Dr. John P. Gray. Complacency, that reposed in every lineament of his face, found expression in his learned but placid address on State Medicine.

Those who believe in a higher medical education, and who expect the commonwealth to further that end, were dejected by what they heard, until Dr. Carroll, of Richmond county, read, upon the same subject, a well-ribbed and well-limbed address, that vibrated with a living spirit and espoused the cause that must yet prevail. Everybody listened to Dr. Carroll, and, with two or three exceptions, everybody liked what he said.

As a rule, the contributions were worthy of the association, which is saying much. The discussion on pneumonia, which was opened by Dr. Austin Flint in his usual charming style, cannot fail to hold the attention of those who read it. It will hardly be read in one evening.

Dr. Simeon T. Clark, of Lockport, will be remembered for his paper on vivisection in pneumonia, and he will not be forgotten for other handsome things. It is cheerful to those living west of the Alleghanies, (which range is said to separate those who write well from those who practice well,) that one of their number can write as well as work.

The western end of the State was well represented in numbers, not to speak of ability; and the great cities of New York and Brooklyn were represented well—although not well represented.

Those fellows who were not "too much overcome by the hospitalities of the people," visited the Carnegie Laboratory, and in the library there saw another instance of what a great, good and persistent man can do. The strong and true efforts of Dr. Gouley have accomplished for the State Association many things besides a library; but he still labors untiringly, and the warmth that radiates from him, thaws indifference when it ventures near.

There are two classes, it appears, who attend State medical meetings: one goes to learn, while the other goes to laugh; and it, therefore, follows that in order to please everybody, there must be set apart time for diversion, as well as for discussion. On this occasion, there were morning, afternoon and evening sessions for work, and only single, short, night sessions for amusement.

Nearly everybody observed the schedule for the first day, and rested the next.

It is understood that there will be a shorter programme the coming year; and some one proposed that only one doctor be permitted to make a speech at the banquet, and that he be "put on the limits." This arrangement would make the banquet (to use the language of the patent medicine editor,) "not only palatable, but tolerated by the most sensitive stomachs."

The hotel corridors must have been interesting to the lay observer. How doctors can talk! There was one thirty-five-year-old bore arguing against the use of anæsthetics in labor, basing his remarks upon a personal experience in the care of three thousand cases, all of which, *mirabile dictu*, recovered, and none of them took an anæsthetic. Then there was the man who disbelieved in the identity of diphtheria and croup, and there also was the other man who (God help us,) was satisfied that they were the same thing.

A doctor who thinks himself wiser than his neighbor, is only truly wise when his neighbor knows it.

PREPOSITION.

